# Enhancing Stress Corrosion Cracking Resistance of Low Cu-Containing Al-Zn-Mg-Cu Alloys by Aging Treatment Control

**DOI:** 10.3390/ma17235678

**Published:** 2024-11-21

**Authors:** Ying Li, Mingyang Yu, Xiwu Li, Kai Wen, Lizhen Yan, Kai Zhu, Wei Xiao

**Affiliations:** 1 State Key Laboratory of Nonferrous Metals and Processes, GRINM Group Co., Ltd., Beijing 100088, China; lixiwu@grinm.com (X.L.); wenkai@grinm.com (K.W.); yanlizhen@grinm.com (L.Y.); zhukai@grinm.com (K.Z.); xiaowei@grinm.com (W.X.); 2 GRIMAT Engineering Institute Co., Ltd., Beijing 101407, China; 3 General Research Institute for Nonferrous Metals, Beijing 100088, China

**Keywords:** Al-Zn-Mg-Cu alloy, stress corrosion cracking, over-aging, microstructure, mechanical properties

## Abstract

The 7085 aluminum alloy with a low Cu content is an important lightweight structural material in the aerospace field due to its advantages of low density, high specific strength, and high hardenability. However, like other high-strength Al-Zn-Mg-Cu alloys, this alloy is susceptible to stress corrosion cracking (SCC). Additionally, the lower Cu content may increase its tendency toward SCC, potentially impacting the safe use of this alloy. Therefore, this study investigated the effects of aging treatment processes on the mechanical properties and SCC resistance of 7085 aluminum alloy. And the factors affecting the properties of the alloy were analyzed by optical microscope (OM), scanning transmission electron microscope (STEM), and energy-dispersive X-ray spectroscopy (EDS) analyses. The results indicated that increasing the secondary aging temperature and adding a tertiary aging step can significantly reduce the alloy’s susceptibility to SCC while meeting the mechanical performance requirements. The reduced SCC sensitivity was mainly attributed to the increased spacing of grain boundary precipitates, a wider precipitate-free zone at the grain boundaries, and a higher Cu content in the grain boundary precipitates.

## 1. Introduction

Al-Zn-Mg-Cu aluminum alloys (also known as 7xxx series alloys) are high-strength, heat-treatable aluminum alloys that are widely used as large structural components in aerospace, rail transportation, and defense equipment due to their high specific strength, low density, good toughness, and excellent overall performance [1,2]. However, their susceptibility to stress corrosion has been a major focus of research for scholars, manufacturers, and end-users.

To address the issue of stress corrosion in 7xxx series aluminum alloys, researchers have conducted extensive studies. Three primary mechanisms of stress corrosion cracking (SCC) were identified. They are the anodic dissolution theory, the hydrogen-induced cracking theory, and a mixed mechanism involving both anodic dissolution and hydrogen-induced cracking [3]. Prior to the 1970s, the anodic dissolution theory was the dominant explanation for the SCC mechanisms in aluminum alloys. This theory posits that the difference in electrochemical potential between the grain boundary precipitates and the precipitate-free zones or the matrix causes preferential dissolution of the precipitates at the grain boundaries. Thus, it results in crack formation along grain boundaries and leads to electrochemical corrosion cracking. However, some aspects of the anodic dissolution process, such as its exact mechanisms and formation process, remain unclear and contentious. In 1969, Grubl and his colleagues first observed that high-strength aluminum alloys could exhibit hydrogen-induced loss of ductility, leading to a growing interest in the hydrogen-induced cracking theory [4].

To improve the stress corrosion resistance of 7xxx series aluminum alloys, researchers have focused on optimizing alloy composition, deformation, and heat treatment processes. Adding elements such as Cu, Mn, and Cr to Al-Zn-Mg alloys significantly enhances their resistance to stress corrosion. The addition of Cu, for example, optimizes the grain boundary precipitates and the precipitate-free zones, reducing the potential difference within and along grain boundaries, thereby improving corrosion resistance [5,6,7]. Mg tends to segregate at grain boundaries, where free Mg forms “Mg-H” complexes with hydrogen, increasing hydrogen solubility at the boundaries and promoting boundary embrittlement. An increased Zn/Mg ratio narrows the precipitate-free zones at grain boundaries and reduces the Zn content in the aluminum matrix, which can heighten the alloy’s susceptibility to stress corrosion [8]. Excess impurities such as Fe and Si can form coarse compounds within the alloy, such as Al_7_Cu_2_Fe, Mg_2_Si, and (Fe, Mn)Al_6_. These coarse compounds are prone to fracture and create voids under low stress, adversely affecting corrosion resistance [9,10].

In addition to optimizing alloy composition, researchers have improved corrosion resistance in 7xxx series aluminum alloys by refining the grain structures and precipitates through adjustments in deformation processes and heat treatments [11]. Song [12] et al. found that a lower cooling rate allows sufficient time for Cu diffusion, enhancing the Cu content in grain boundary precipitates of the AA7050 alloy and thus improving its corrosion resistance. Similarly, Xie [13] et al. employed a step quenching technique to increase the size and spacing of grain boundary precipitates in the 7097 alloy. Meanwhile, the Cu content in these precipitates was increased. As a result, the corrosion resistance of this low-Cu alloy was enhanced. Aging treatments also impact corrosion performance by altering the precipitate’s characteristics. For example, T6 single-stage aging imparts high mechanical strength to 7xxx aluminum alloys, but the continuous distribution of precipitates at the grain boundaries significantly reduces corrosion resistance. To improve this, researchers have developed two-stage aging and retrogression and re-aging (RRA) heat treatments [14]. The T7X tempers (over-aging treatments, such as T76, T74, and T73) were also designed to increase SCC resistance but generally result in a 10–15% loss in alloy strength [15]. In 1989, Alcoa introduced the RRA treatment, which significantly enhanced the comprehensive performance of 7xxx aluminum alloys; these improvements are closely tied to the alloy’s microstructural changes. During the aging process, precipitates in 7xxx alloys typically evolve from the GP zones to the η’ (MgZn_2_) phase and finally to the η (MgZn_2_) phase. The retrogression stage in RRA promotes the coarsening of η (MgZn_2_) precipitates at the grain and sub-grain boundaries while maintaining a fine η’ distribution within grains. These coarse η precipitates act as hydrogen traps, preventing hydrogen atoms from concentrating near grain boundaries, thus reducing SCC susceptibility. RRA also alters Cu and Mg enrichment at the grain boundaries, reduces dislocation density, and further lowers SCC sensitivity [16]. However, due to the short retrogression time (less than 1 h), RRA is not suitable for thick-section aluminum alloy products [17].

The 7085 aluminum alloy is one type of 7xxx series alloy. Compared to other high-strength 7xxx aluminum alloys, it has a lower Cu content. This alloy is widely used in wing spars and rib components due to its low quench sensitivity and high strength and toughness [18]. However, alloys with a low Cu content are more susceptible to SCC. While studies have been conducted on the SCC behavior of low-Cu 7xxx alloys, the mechanisms underlying SCC formation are not yet fully understood. And SCC issues still arise in practical applications. Therefore, this study systematically investigates SCC in the 7085 aluminum alloy, aiming to reduce SCC susceptibility by optimizing heat treatment processes while ensuring that the alloy’s mechanical properties meet application requirements. The findings of this research would provide theoretical guidance for addressing SCC issues with 7085 aluminum alloy in real-world engineering applications.

## 2. Materials and Methods

### 2.1. Materials and Aging Treatment

The material used in this study was a rolled 7085 aluminum alloy plate with a thickness of 160 mm, provided by Southwest Aluminum (Group) Co., Ltd., Chongqing, China. The chemical compositions were composed of 7.50 Zn, 1.50 Mg, 1.67 Cu, 0.11 Zr, 0.04 Fe, 0.02 Si, and balance Al, in wt%. The chemical composition of the 7085 aluminum alloy was tested according to the standard GB/T 20975-2020 [19]. The samples used for mechanical property, corrosion tests, and microstructure investigation were cut from near the mid-thickness of the plate. All samples were then solution-treated for 1 h at 476 °C, followed by quenching to room temperature and artificial at different temperatures. The detailed aging treatments are shown in Table 1.

### 2.2. Electrical Conductivity Tests

The electrical conductivity of the samples in different aging treatments was tested by a conductivity meter (Foerster Sigmatest-2.069, Reutlingen, Germany) at room temperature. The measurement frequency was 120 kHz, and the accuracy of the measurement was ±0.5%.

### 2.3. Mechanical Property Tests

**Figure 1:**
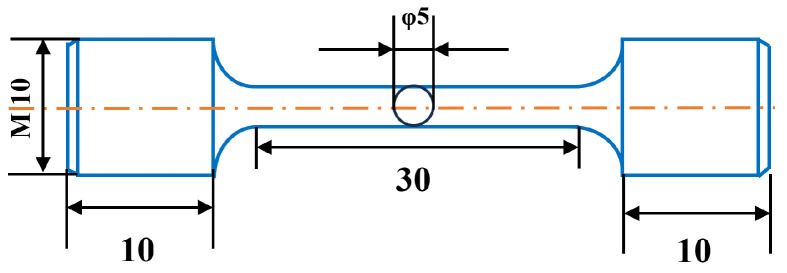
Diagram of dimensions of the tensile samples.

The tensile samples in different aging states were tested at a constant crosshead speed of 2 mm/min using an electronic universal testing machine (MTS WD-3100, Norwood, MA, USA) at room temperature. For each condition, at least three samples were used to calculate average values. The tensile samples were taken at the mid-thickness of the plate along the rolling direction. The dimensions of the tensile samples are shown in Figure 1.

### 2.4. Corrosion Tests

**Figure 2:**
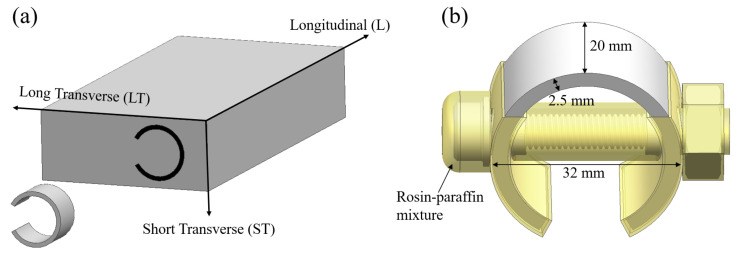
Diagram of extracted position and dimensions of the C-ring specimens: (**a**) extracted position; (**b**) dimensions of the C-ring specimens.

The corrosion tests were carried out in C-ring stress corrosion and slow strain rate tensile testing (SSRT). The C-ring specimens were sampled at the mid-thickness in the short transverse (ST) direction of the plate (Figure 2a). The size of the C-ring specimens was decided to be 20 mm wide, 32 mm in the outer diameter, and 2.5 mm thick (Figure 2b). The surface of the samples needs to be machined to a bright finish. Moreover, the samples were protected using a rosin–paraffin mixture with a mixing ratio of 1:5, avoiding the occurrence of crevice and galvanic corrosion between the C-ring samples and the stressing bolt (Figure 2b). The load stress on the samples was 241 MPa. The C-ring SCC tests were carried out in the 3.5% wt% NaCl solution. In the stress corrosion test, new equipment was exploited to maintain the air temperature at 35 °C and the relative humidity controlled at 45 ± 10%, as well as a mild air circulation throughout the entire test cycle. In addition, specimens were totally immersed in the salt solution for 10 min of each hour and then removed from the solution to dry for 50 min. The abovementioned periodical SCC test was carried out for 20 days, and then all the samples were taken out and prepared for microstructural observation.

The slow strain rate tensile (SSRT) test was carried out in silicone oil and 3.5% NaCl solution at a 35 °C temperature, with a strain rate of 10^−6^ S^−1^. The SSRT samples were also extracted from the mid-thickness of the plate.

### 2.5. Microstructural Investigations

The corrosion cracking features were observed by optical microscopy (OM, Zeiss Axiovert 200 MAT, Oberkochen, Germany). The morphology and composition of the precipitates in the large grain boundaries of different aging samples were investigated using a scanning transmission electron microscope (STEM, Talos F200X G2, Houston, TX, USA) combined with energy-dispersive X-ray spectroscopy (EDS). The samples were prepared by twin-jet thinning in a solution comprising 25 vol.% nitric acid and 75 vol.% methanol at −30 °C and 14 V. For the EDS analysis of grain boundary precipitates (GPBs), each data point was the arithmetic mean of at least 10 measured GBPs obtained at 5 different grain boundaries.

## 3. Results

### 3.1. Electrical Conductivity and Tensile Properties

**Figure 3:**
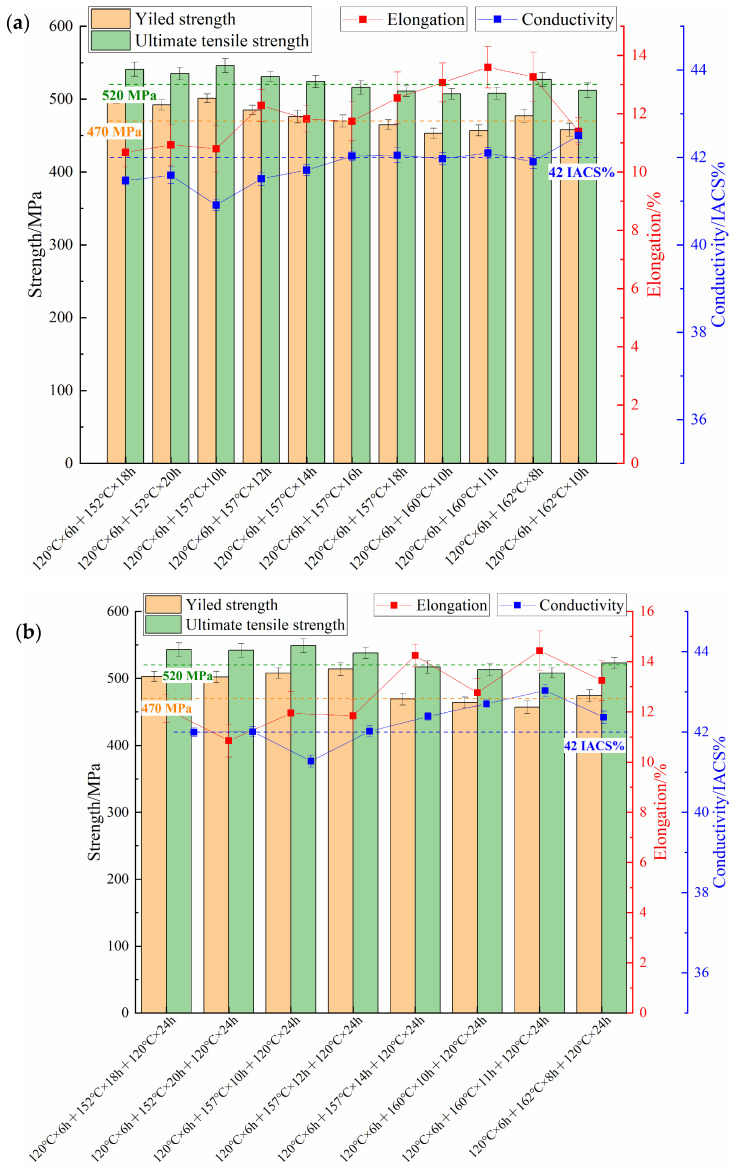
Tensile properties and conductivity of samples after different aging treatments: (**a**) secondary aging treatment; (**b**) third-stage aging treatment.

Figure 3 shows the characteristics of tensile strength and electrical conductivity changes in the 7085 aluminum alloy under different aging conditions. Under two-step aging conditions, with a constant secondary aging temperature, the tensile strength of the 7085 aluminum alloy decreases as the aging time increases while electrical conductivity increases. When the aging time is constant, increasing the secondary aging temperature similarly results in a decrease in tensile strength and an increase in electrical conductivity (Figure 3a). According to AMS4414 standards, the minimum yield strength, ultimate tensile strength, and elongation for 160 mm thick 7085-T7452 aluminum alloy forgings for engineering applications are 441 MPa, 490 MPa, and 9%, respectively. For safety in service conditions, the actual performance of the materials typically exceeds these standards, with a minimum yield strength, ultimate tensile strength, and elongation of 470 MPa, 520 MPa, and 9%, respectively, required for safe applications.

In practical use, not only must the alloy meet mechanical property requirements, but its stress corrosion resistance must also be adequate. For the 7085 aluminum alloy forgings with acceptable SCC resistance, the conductivity is typically no less than 42% IACS, setting this as a minimum conductivity standard. Analysis of the tensile properties and conductivity under two-step aging conditions revealed that no specimens met the minimum requirements for tensile properties and conductivity. Thus, two-step aging alone is insufficient to meet the toughness and SCC resistance standards for the 7085 aluminum alloy in this study. Consequently, this study explored three-step aging to optimize both the mechanical and corrosion-resistant properties of the alloy.

Figure 3b illustrates the characteristics of the tensile properties and electrical conductivity of the alloy under various three-step aging conditions. Compared to two-step aging, the addition of a third aging stage at 120 °C for 24 h increased the alloy’s conductivity by 0.5–1 IACS%, with a slight reduction in tensile strength and minimal change in elongation. Specifically, the alloy treated with three-stage aging at 120 °C for 6 h, 162 °C for 8 h, and a final stage of 120 °C for 24 h achieved a yield strength of 474 MPa, an ultimate tensile strength of 523 MPa, an elongation of 13.25%, and a conductivity of 42.37 IACS%, meeting the requirements for practical applications.

### 3.2. Stress Corrosion Cracking Properties

To further analyze the impact of different aging treatments on the stress corrosion performance of the alloy, samples treated under two typical aging conditions—120 °C for 6 h followed by 152 °C for 18 h (120 °C × 6 h + 152 °C × 18 h), and 120 °C for 6 h followed by 162 °C for 8 h, and finally, 120 °C for 24 h (120 °C × 6 h + 162 °C × 8 h+120 °C × 24 h)—were selected for stress corrosion performance testing and microstructural analysis.

**Figure 4:**
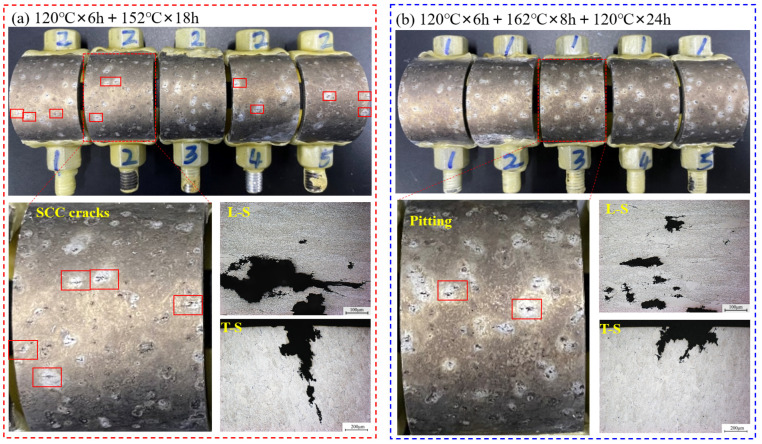
Macroscopic morphology and crack growth features of C-ring samples in different aging treatments: (**a**) aging at 120 °C × 6 h+152 °C × 18 h; (**b**) aging at 120 °C × 6 h + 162 °C × 8 h + 120 °C × 24 h.

Figure 4 shows the macroscopic appearance of the C-ring stress corrosion samples and their corrosion characteristic micrographs under the two types of aging conditions. The results show that after treatment at 120 °C for 6 h followed by 152 °C for 18 h, the alloy exhibited significant SCC upon testing, with the cracks primarily initiating near recrystallized grains and propagating along high-angle grain boundaries (Figure 4a). In contrast, the alloy treated with the three-step aging process (120 °C for 6 h, 162 °C for 8 h, and 120 °C for 24 h) did not show any noticeable SCC on the sample surface after testing; instead, pitting corrosion was predominantly observed. Further metallographic analysis confirmed that this corrosion feature was indeed pitting, primarily occurring near the recrystallized grains (Figure 4b). The C-ring stress corrosion test results show that the stress corrosion resistance of the alloy is significantly improved by the three-step aging treatments of 120 °C for 6 h, 162 °C for 8 h, and 120 °C for 24 h.

**Figure 5:**
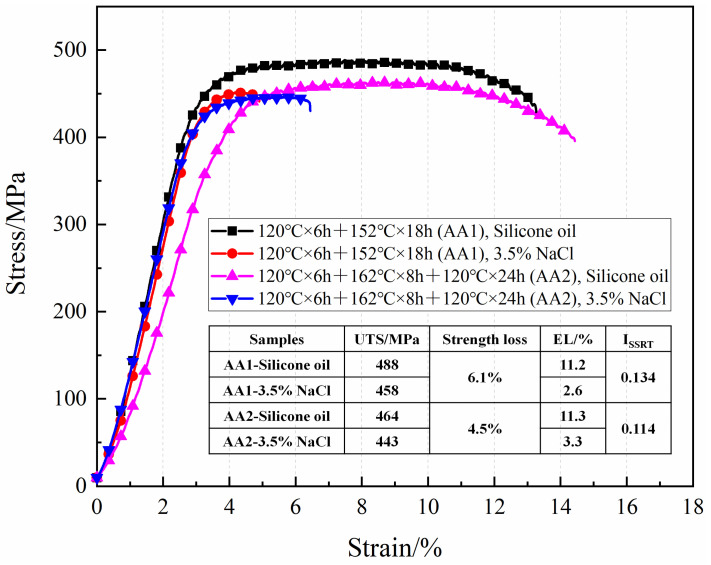
SSRT engineering stress–strain curves of samples after different aging treatments tested in silicone oil and 3.5% NaCl conditions.

Figure 5 presents the engineering strain–stress curves for the SSRT samples aged under the conditions of 120 °C × 6 h + 152 °C × 18 h and 120 °C × 6 h + 162 °C × 8 h + 120 °C × 24 h, tested in 35 °C silicone oil and 35°C 3.5% NaCl solution conditions. For the samples treated with the 120 °C × 6 h + 152 °C × 18 h aging process, the ultimate tensile strength in the 3.5% NaCl condition decreased from 488 MPa under inert conditions to 458 MPa, indicating a strength loss of 6.1%. Additionally, the elongation dropped significantly from 11.2% under inert conditions to 2.6%. In the case of samples aged with the three-step treatment (120 °C × 6 h + 162 °C × 8 h + 120 °C × 24 h), the ultimate tensile strength decreased from 464 MPa under inert conditions to 443 MPa in the 3.5% NaCl solution, representing a strength loss of 4.5%. The elongation also reduced from 11.3% under inert conditions to 3.3%.

The SSRT intensity of the alloy is calculated using Formula (1):

(1) $$I_{SSRT}=1-\frac{\sigma_{fw}\cdot \left(1+\delta_{fw}\right)}{\sigma_{fA}\cdot \left(1+\delta_{fA}\right)}$$

In the formula:

*σ_fw_*—is the tensile strength of the sample in the corrosive medium, N/mm^2^;
*σ_fA_*—is the tensile strength of the sample in the inert medium, N/mm^2^;
*δ_fw_*—is the percentage of elongation at break of the sample in the corrosive medium, %;
*δ_fA_*—is the percentage of elongation at break of the sample in the inert medium, %.

The calculated results show that the *I_SSRT_* of the samples under the aging conditions of 120 °C for 6 h and 152 °C for 18 h is 0.134, which is higher than the value of 0.114 for the samples subjected to the aging conditions of 120 °C for 6 h, 162 °C for 8 h, and 120 °C for 24 h. The results further indicate that the stress corrosion resistance of the alloy is significantly improved after the three-step aging treatment of 120 °C for 6 h, 162 °C for 8 h, and 120 °C for 24 h.

### 3.3. Microstructures

**Figure 6:**
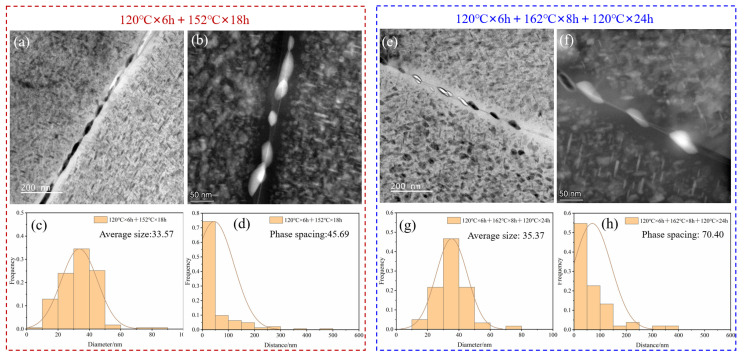
TEM images and distribution diagrams of GBP sizes and spacings for samples under different aging conditions: (**a**,**b**) TEM images of GBPs in samples aged at 120 °C × 6 h + 152 °C × 18 h; (**c**) size distribution diagram of GBPs in samples aged at 120 °C × 6 h + 152 °C × 18 h; (**d**) spacing distribution diagram of GBPs in samples aged at 120 °C×6h+152 °C × 18 h; (**e**,**f**) TEM images of GBPs in samples aged at 120 °C × 6 h + 162 °C × 8 h + 120 °C×24 h; (**g**) size distribution diagram of GBPs in samples aged at 120 °C × 6 h + 162 °C × 8 h + 120 °C × 24 h; (**h**) spacing distribution diagram of GBPs in samples aged at 120 °C × 6 h + 162 °C × 8 h + 120 °C × 24 h.

The characteristics of GBPs are the main factors affecting the stress corrosion performance of the alloy. To explore the reasons for the differences in SCC of the samples under different aging conditions, the GBP characteristics of the samples subjected to the aging treatments of 120 °C × 6 h + 152 °C × 18 h and 120 °C × 6 h + 162 °C × 8 h + 120 °C × 24 h were observed. The results are shown in Figure 6a,b and Figure 6b,e, respectively. It was observed that the distance of the GBPs in the samples treated with the three-step aging was larger than that in the samples treated with the two-stage aging. Statistical analysis of the size and distance of the GBPs in the two types of aging processes was conducted. The statistical results (shown in Figure 6c,d,g,h) indicate that the average size of the GBPs in the samples after the two-step aging was 33.57 nm, with a precipitate spacing of 45.69 nm. In contrast, the samples after the three-step aging exhibited an average size of 35.37 nm, comparable to that of the two-step aging samples. However, the precipitate spacing in the three-step aging samples was 70.40 nm, significantly larger than that in the two-step aging samples. Additionally, the width of the precipitate-free zone (PFZ) of the grain boundary also increased after the three-step aging treatment.

**Figure 7:**
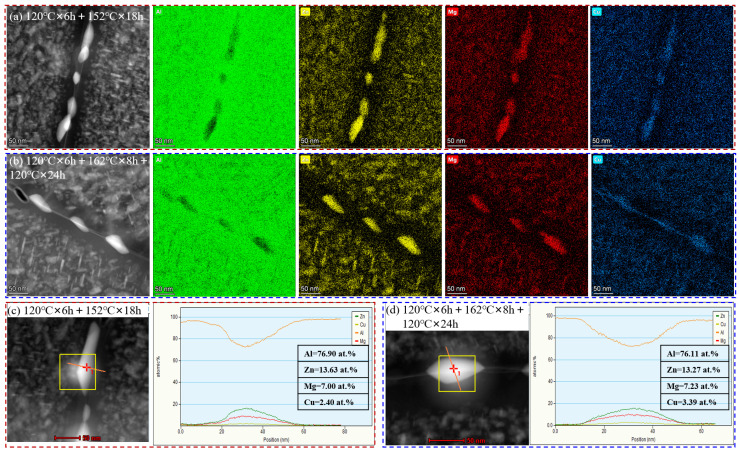
STEM images and EDS analysis of the samples in different aging treatments: (**a**,**c**) aging at 120 °C × 6 h + 152 °C × 18 h; (**b**,**d**) aging at 120 °C × 6 h + 162 °C × 8 h + 120 °C × 24 h.

STEM was used to perform the EDS analysis on the grain boundaries of the samples under two types of aging conditions. Figure 7a,b show the EDS mapping of the samples aging at 120 °C × 6 h + 152 °C × 18 h and 120 °C × 6 h + 162 °C × 8 h + 120 °C × 24 h, respectively. The EDS results show that the GPBs of the samples under the two aging conditions were AlZnMgCu phases. During the aging process, the Al and Cu elements in the matrix will diffuse into the MgZn_2_ phase on the grain boundaries, replacing some Zn atoms to form the Mg(Zn,Al,Cu)_2_ phase. Further point composition analysis of the GPBs (as shown in Figure 7c,d) found that the Cu element content of the GPBs after the three-step aging was higher than that of the two-step aging samples.

## 4. Discussion

In the Al-Zn-Mg-Cu alloys, the characteristics of the precipitates play a crucial factor in influencing the mechanical properties and corrosion resistance of the alloy, particularly under similar conditions of grain structure. The high mechanical properties are typically associated with the fine size and high densities of the precipitates. However, if the precipitates continuously precipitate in the grain boundaries, the susceptibility to stress corrosion cracking would increase significantly. This occurs because, during the aging process, Mg and Zn elements initially enrich at the grain boundaries, forming the MgZn_2_ phase. As the aging time increases, Al and Cu elements from the matrix diffuse toward the MgZn_2_ phase at the grain boundaries, replacing some Zn atoms to form the Mg(Zn, Al, Cu)_2_ phase [20]. The Mg(Zn, Al, Cu)_2_ phases precipitated at the grain boundaries exhibit a lower electrode potential than the aluminum matrix, functioning as anodes and making them more susceptible to corrosion. Once corrosion occurs, it tends to propagate along the grain boundaries, so the more continuous the distribution of precipitated phases at the grain boundaries, the faster the propagation of corrosion cracks [21].

To achieve a discontinuous distribution of GBPs, researchers have employed over-aging processes such as T7X to treat the alloy. However, in this study, the T74 (120 °C × 6 h + 152 °C × 18 h) over-aging heat treatment applied to the 7085 alloy still exhibited significant characteristics of stress corrosion cracking. Extending the aging time or improving the aging temperature in the second stage of aging resulted in a significant reduction in the strength of the alloy, while the change in electrical conductivity was not very pronounced (Figure 3a). The electrical conductivity of alloys is closely linked to their microstructural characteristics. Generally, the larger and fewer the precipitates, the less lattice distortion they cause. This reduces electron scattering, lowers resistivity, and thereby increases the alloy’s electrical conductivity [22]. Thus, the electrical conductivity can be used to indirectly reflect the corrosion resistance of the alloy. Higher conductivity indicates better corrosion resistance. As shown in Figure 3a and Figure 4, controlling the second-stage aging temperature and time does not synergistically optimize the mechanical properties and corrosion resistance of the alloy. Therefore, a three-step aging process was considered to regulate the properties of the alloy.

As shown in Figure 3b, after the three-step aging treatment, the reduction in alloy strength was not significant, but the electrical conductivity showed a notable increase. As shown in Figure 4b, the results from the C-ring stress corrosion tests indicated that after the three-step aging process (120 °C × 6 h + 162 °C × 8 h + 120 °C × 24 h), the corrosion characteristics on the sample surface transitioned from the presence of stress corrosion cracks to pitting corrosion. Additionally, results from the SSRT corrosion tests showed that the stress corrosion sensitivity factor decreased from 0.134 after the two-step aging to 0.114 after the three-step aging process (Figure 5). The abovementioned results indicated that increasing the secondary aging temperature and adding a tertiary aging step can significantly reduce the alloy’s susceptibility to SCC while meeting the mechanical performance requirements.

The precipitates’ features, observed from Figure 6 and Figure 7, show that the spacing of GBPs increased, and the width of the PFZ at the grain boundaries also widened, along with an increase in the Cu content in the GBPs. The increase in the Cu content in the GPBs raised their electrode potential, narrowing the potential difference with the matrix. Moreover, the increased spacing of the GBPs decreased the growth rate of the stress corrosion cracks. This suppression of anodic dissolution and low growth rate of stress corrosion cracks inhibited the initiation and expansion of stress corrosion cracks. Thus, the corrosion resistance of the alloy was improved by increasing the secondary aging temperature and adding a tertiary aging step.

## 5. Conclusions

This study focused on the issue of stress corrosion cracking in 7085 aluminum alloy and investigated the effects of the heat treatment process on the mechanical properties and stress corrosion performance of the alloy. The main conclusions are as follows:

(1) Extending the aging time leads to a decreasing trend in the tensile strength of the alloy, while the electrical conductivity shows an increasing trend during the two-step aging process. Increasing the second-stage aging temperature also results in a decreasing trend in tensile strength and an increasing trend in electrical conductivity.
(2) Compared to the two-step aging process, the three-step aging resulted in an increase in the alloy’s electrical conductivity by 0.5 to 1 IACS%, while the tensile strength slightly decreased, and there was no significant change in elongation. Specifically, the alloy treated with three-step aging at 120 °C for 6 h, 162 °C for 8 h, and a final stage of 120 °C for 24 h achieved a yield strength of 474 MPa, an ultimate tensile strength of 523 MPa, an elongation of 13.25%, and a conductivity of 42.37 IACS%, meeting the requirements for practical applications.
(3) Increasing the secondary aging temperature and adding a tertiary aging step can significantly reduce the SCC sensitivity of the alloy while meeting the mechanical performance requirements. The reduced SCC sensitivity was mainly attributed to the increased spacing of GPBs, a wider PFZ at the grain boundaries, and a higher Cu content in the grain boundary precipitates.

## Figures and Tables

**Table 1:** Aging conditions used for 7085 aluminum alloy samples.

Number	First-Step Aging	Second-Step Aging	Third-Step Aging
1	120 °C, 6 h	152 °C, 18/20 h	-
2		152 °C, 18/20 h	120 °C, 24 h
3		157 °C, 10/12/14/16/18 h	-
4		157 °C, 10/12/14 h	120 °C, 24 h
5		160 °C, 10/11	-
6		160 °C, 10/11	120 °C, 24 h
7		162 °C, 8/10	-
8		162 °C, 8/10	120 °C, 24 h

## Data Availability

The raw/processed data required to reproduce these findings cannot be shared at this time, as the data also comprise a part of an ongoing study.
